# Inflamed Eyes

**Published:** 1894-02-10

**Authors:** R. Lawford Knaggs

**Affiliations:** Ophthalmic Surgeon to the Leeds Public Dispensary


### MEDICAL PROGRESS AND HOSPITAL CLINICS.

[*The Editor will be glad to receive offers of co-operation and contributions from members of the profession. All letters should be addressed to* THE EDITOR, THE LODGE, PORCHESTER SQUARE, LONDON, W.]

### INFLAMED EYES.

By R. LAWFORD KNAGGS, M.A., M.C., Cantab, F.R.C.S., Ophthalmic Surgeon to the Leeds Public Dispensary.

The object of this paper is to show how the more common conditions met with in practice as inflamed eyes can be distinguished from one another. In the large majority of instances the various inflammatory conditions can be recognised rapidly and with certainty by simple inspection only.

To the beginner, or to those who, without the advantage of an ophthalmic training, try to acquire some knowledge of eye disease from the material at their disposal, it is difficult to over-estimate the importance of method. Method, to the self-instructor, is invaluable, but in the expert it is developed almost to an unconscious instinct. Let it at once be understood, then, that methodical examination and observation is the keynote of all that follows.

When examining an inflamed eye the surgeon's mind should be busily employed in noting the conditions present, for the diagnosis is to be made from what is seen, and not from what the patient tells him. In making these mental observations a systematic plan of examination will simplify matters and frequently prevent an important factor being overlooked; and so, all those parts of the eye and its accessories, that can be seen, should in turn be examined as far as possible in each individual case. They are:

1. The lids, the eyelashes, and their direction; the lachrymal puncta, and the condition of the lachrymal sac.
2. The conjunctiva, both that lining the lids and that covering the sclerotic, and of course the underlying sclerotic itself.
3. The cornea.
4. The aqueous humor, or the contents and appearance of the anterior chamber.
5. The iris.
6. The pupil.
7. The lens.

And to these may be added:

8. The estimation of tension.

The changes found in one or more of these structures are usually sufficiently obvious, and demonstrate that almost every inflamed eye belongs to one of the following conditions:

(*a*) Inflammatory states of the lids, their hair follicles or their glands.
(*b*) Inflammatory states of the lachrymal apparatus.
(*c*) Inflammations of the conjunctiva.
(*d*) Inflammations of the cornea with or without ulceration.
(*e*) Inflammations of the iris.
(*f*) Glaucoma.

Groups *a* and *b* may be dismissed at once from consideration, for their nature is usually clear, and they have only been included here for the sake of completeness and the illustration of method. In the remaining four groups too much importance cannot be attached to a discriminating diagnosis. Those conditions that are of comparatively lesser moment belong either to the conjunctival or corneal inflammations, but serious forms of disease occur in each of the four categories—sometimes so serious that vision may be completely destroyed, even when carefully treated. Between these extremes are many intermediate phases, and in not a few instances the retention of an eye, as a more or less useful organ of sight, depends upon the early recognition of the nature of the inflammation and the prompt exhibition of proper modes of treatment. This alone would call for care in diagnosis, but the frequency with which different forms of inflamed eyes are confounded, to the prejudice alike of the patient and the practitioner, sufficiently attests the importance of knowing how to go to work to form those clear ideas which are so necessary to the formation of a correct opinion.

And how is this to be done? Simply by noting:

1. The departure from the natural appearance or function in any of the various structures that have been enumerated.
2. The persistence of health in such of them as are unaffected.

The former are the positive signs of the particular inflammation; the latter are the negative indications, and are of great value, because they enable other forms of inflammation to be excluded with certainty.

For instance, a patient seeks advice because his eyes are inflamed. There is obvious photophobia, and on careful inspection of the eyes the conjunctiva covering the sclerotic is seen to be intensely congested, and covered with numerous vessels, which in health are invisible, and that lining the lid is deep red and velvety instead of pale and glistening. There is also more or less evidence of discharge in the recesses of the lids or about their edges. Though these facts point strongly to an inflammation of the conjunctiva, yet the diagnosis is not complete until the cornea is noted to be clear and bright, the iris not to be discoloured, and the pupil readily capable of contraction to light. So the cornea and iris are known to be healthy, and when the tension of the eyes has been estimated and found to be normal—a point which will call for further explanation subsequently—the absence of glaucoma is ascertained.

Thus a diagnosis of conjunctivitis is made partly from the evidences of inflammation in that membrane, but largely by those signs which show that the adjacent structures are not implicated.

The value of exclusion in the diagnosis of inflamed eyes is again illustrated in the next paragraph. Another patient may have the more striking symptoms just described, viz., intolerance of light and intense conjunctival congestion; but when the cornea comes to be examined, it shows a departure from its natural condition in the presence of one or more white spots, or a distinct ulceration of its surface, or a cloudy haze within its substance, and possibly vessels may be seen ramifying upon it or in it. In addition to the corneal blemish it will usually be possible to make out a ring of deeper vascularity immediately surrounding the cornea. This ring, known as "ciliary congestion," and composed of numerous minute parallel straight vessels, is readily seen in most cases where either the cornea or the iris is inflamed, provided there is no increased vascularity of the conjunctiva, but if the latter is present, it may escape detection if not looked for.

These are the positive signs by which an inflammation of the cornea is recognised, but again the activity of the pupil to light, as well as the appearance of the iris itself must be noted, if the cornea is transparent enough to permit it. Should the pupil be sluggish or fixed, it will point to the inflammation affecting the iris, as well as the cornea, a combination that is not unfrequently seen (kerato-iritis); and it is equally essential that the tension should be tested, for the hazy cornea of acute glaucoma might possibly be mistaken for an inflammation of the cornea if its real nature were not cleared up in this way.

In another group of cases "ciliary congestion" is the most prominent feature. Though the conjunctiva may be slightly congested, the question at once arises,

Is it a sign of keratitis, or of iritis, or of both? Now, in the group that is being described, the cornea is seen to be bright and clear, and its surface smooth, so keratitis is out of the question. But the aqueous humor may possibly be turbid, and the colour and appearance of the iris will be altered, and if one eye only be involved, it will show a marked contrast to the other in these as well as in another particular soon to be described. The alteration in appearance is manifested by a blurring of the delicate structural details which are so distinctly defined in an uninflamed iris. Sometimes little masses of lymph are seen to form nodular swellings at the margin of the pupil, and the pupillary space may be encroached upon by them or by little pigmented processes, which, appearing from the posterior surface of the iris, rest upon the anterior surface of the lens, and foretell the existence of pupillary adhesions.

But the alteration extends not only to the colour and appearance, but to the function of the iris. The pupil no longer acts readily to light, but slowly, sluggishly, or not at all, and if dilation is produced by the use of atropine, tags of iris are left attached to the lens surface, so that the form of the dilated pupil is irregular.

Such are the objective signs by which iritis can be recognised, but even in these cases the tension may be of importance, for glaucoma may complicate iritis.

But the most serious of the commoner forms of "inflamed eyes" that are seen in practice still remains to be described. The patient, especially if he belongs to the poorer classes, may, when he first comes for advice, have an eye that is acutely inflamed. The conjunctiva may be intensely congested. The cornea is hazy and dim. If it be stroked with the point of a camel's hair brush it is much more tolerant than its fellow; in other words, it is partially anæsthetic. The corneal haze is not, as a rule, sufficient to present a bar to further examination. The pupil can be seen to be dilated, and oval rather than round. Instead of being black it has a greenish yellow tinge, and it is motionless on exposure to the light. The iris in many cases seems pressed forwards from behind towards the cornea, causing the anterior chamber to appear shallow. All these facts taken together—the conjunctival congestion, the steamy and insensitive cornea, the shallow anterior chamber, the advanced iris, the dilated, oval, and fixed pupil, and its abnormal colour—are sufficient to justify a feeling of moderate certainty as to the nature of the disease, especially if the patient has reached or passed the middle period of life. But to put the finishing touch to the examination the tension of both eyes must be tested. The patient should be told to look down, and then the examiner, placing the tips of his fore-fingers upon the upper lid, exerts a gentle and alternating pressure upon the eyeball, and carefully notes the sensation of greater or less tightness that it presents. It will then be found that the tension of the inflamed eye is higher than is natural, and there need no longer be any hesitation in describing the condition as glaucoma. In a case in which the conditions are so marked as are here supposed, the eyeball may be so hard as to be almost incapable of being dimpled by the finger tips, but very frequently an increase of tension much less obvious than this has to suffice for the confirmation of the diagnosis.

There is no desire on the part of the writer to minimize the importance of the pain, which in acute glaucoma is felt in the eye, the temple, and the nose, or the rapid failure of vision which in a very short time—estimated by days or even hours—may diminish to a mere perception of light. Indeed, it is these symptoms, together with the inflammation, that force the patient to seek advice. But an attempt has been made to show how much can be made out without invoking the subjective sensations of the patient, and the reader's attention has been particularly directed to those signs which can be seen and felt by the surgeon, and which, as in the other conditions that have been described, are quite sufficient for the recognition of the disease.

A consideration of methods of treatment is outside the scope of the present paper. The frequent and disastrous mistakes committed by many who have not grasped the principles that underlie correct differentiation constitute an eloquent commentary on the dangers of the indiscriminate or heedless management of inflamed eyes.

Whoever has carefully worked out his cases point by point, until he can with certainty distinguish between inflammation of the conjunctiva, of the cornea, and of the iris, and not mistake glaucoma for one or all of them, can easily refer to the subject in his text-book and select the treatment that seems most applicable to any particular case. But to such as are incapable of separating one inflamed eye from another a text-book is a hidden mystery. The pages devoted to iritis, glaucoma, possibly cataract and choroiditis may be scanned without knowledge and without understanding, and, after much apparent labour, a treatment suited to one disease is apt to be carefully selected and applied to another.

In this, as in all branches of knowledge, a good and securely laid foundation is necessary if the experience that is built upon it is ever to be a solid and substantial superstructure.

